# Crystal structure of *N*-[3-(di­methyl­amino)­prop­yl]-*N*′,*N*′,*N*′′,*N*′′-tetra­methyl-*N*-(*N*,*N*,*N*′,*N*′-tetra­methyl­form­amid­in­ium­yl)guanidinium bis­(tetra­phenyl­borate)

**DOI:** 10.1107/S2056989015023336

**Published:** 2015-12-12

**Authors:** Ioannis Tiritiris, Willi Kantlehner

**Affiliations:** a Fakultät Chemie/Organische Chemie, Hochschule Aalen, Beethovenstrasse 1, D-73430 Aalen, Germany

**Keywords:** crystal structure, bis­amidinium salt, tetra­phenyl­borate, C—H⋯π inter­actions

## Abstract

In the title salt, C_15_H_36_N_6_^2+^·2C_24_H_20_B^−^, the three N—C bond lengths in the central C_3_N unit of the bis­amidinium ion range between 1.388 (3) and 1.506 (3) Å, indicating single- and double-bond character. Furthermore, four C—N bonds have double-bond character. Here, the bond lengths range from 1.319 (3) to 1.333 (3) Å. Delocalization of the positive charges occurs in the N/C/N and C/N/C planes. The dihedral angle between both N/C/N planes is 70.5 (2)°. In the crystal, C—H⋯π inter­actions between H atoms of the cation and the benzene rings of both tetra­phenyl­borate ions are present. The benzene rings form aromatic pockets, in which the bis­amidinium ion is embedded. This leads to the formation of a two-dimensional supra­molecular pattern along the *ab* plane.

## Related literature   

For the synthesis of similar salts to the title compound, see: Bauer *et al.* (1968 ▸). For the crystal structure of *N*,*N*,*N*′,*N*′-tetra­methyl­chloro­formamidinium chloride, see: Tiritiris & Kantlehner (2008 ▸). For the crystal structures of alkali metal tetra­phenyl­borates, see: Behrens *et al.* (2012*a* ▸). For the synthesis of *N*′′-[3-(di­methyl­amino)­prop­yl]-*N*,*N*,*N*′,*N*′-tetra­methyl­guanidine, see: Tiritiris & Kantlehner (2012*b* ▸). For the crystal structure of *N*,*N*,*N*′,*N*′-tetra­methyl-*N*′′-[3-(tri­methyl­aza­nium­yl)prop­yl]guanidinium bis­(tetra­phenyl­borate) acetone disolvate, see: Tiritiris (2013*a* ▸). For the crystal structure of *N*-[3-(di­methyl­amino)­prop­yl]-*N*,*N*′,*N*′,*N*′′,*N*′′-penta­methyl­guanidinium tetra­phenyl­borate, see: Tiritiris (2013*b* ▸). For the use of intensity quotients and differences in absolute structure refinement, see: Parsons *et al.* (2013 ▸).
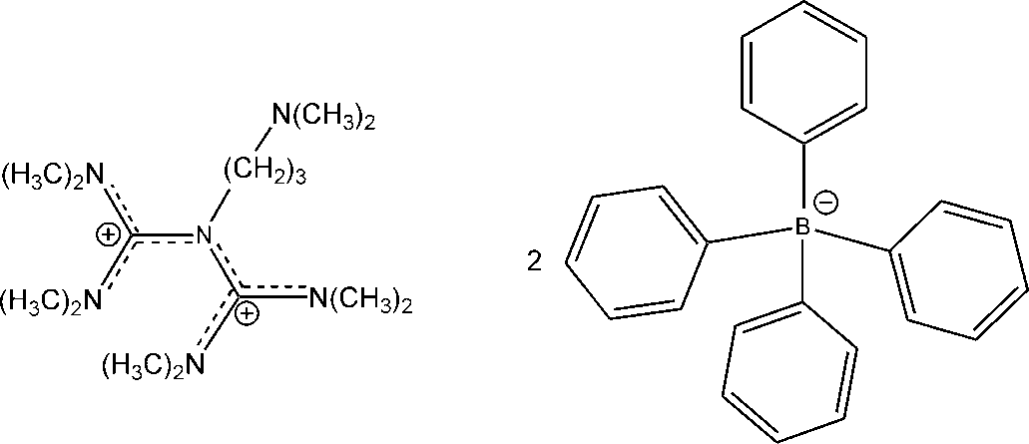


## Experimental   

### Crystal data  

C_15_H_36_N_6_^2+^·2C_24_H_20_B^−^
*M* *_r_* = 938.92
Monoclinic, 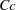

*a* = 17.1964 (5) Å
*b* = 17.6641 (7) Å
*c* = 17.4751 (6) Å
β = 98.752 (1)°
*V* = 5246.4 (3) Å^3^
*Z* = 4
Mo *K*α radiation
μ = 0.07 mm^−1^
*T* = 100 K
0.22 × 0.18 × 0.15 mm

### Data collection  

Bruker–Nonius KappaCCD diffractometer
11667 measured reflections
11660 independent reflections
10531 reflections with *I* > 2σ(*I*)
*R*_int_ = 0.040

### Refinement  

*R*[*F* ^2^ > 2σ(*F* ^2^)] = 0.038
*wR*(*F* ^2^) = 0.087
*S* = 1.06
11660 reflections
651 parameters
2 restraints
H-atom parameters constrained
Δρ_max_ = 0.21 e Å^−3^
Δρ_min_ = −0.20 e Å^−3^
Absolute structure: Flack *x* determined using 4294 quotients [(*I* ^+^)−(*I* ^−^)]/[(*I* ^+^)+(*I* ^−^)] (Parsons *et al.*, 2013 ▸)
Absolute structure parameter: −0.8 (8)

### 

Data collection: *COLLECT* (Hooft, 2004 ▸); cell refinement: *DENZO-SMN* (Otwinowski & Minor, 1997 ▸); data reduction: *DENZO-SMN*; program(s) used to solve structure: *SHELXS97* (Sheldrick, 2008 ▸); program(s) used to refine structure: *SHELXL2014* (Sheldrick, 2015 ▸); molecular graphics: *DIAMOND* (Brandenburg & Putz, 2005 ▸); software used to prepare material for publication: *SHELXL2014*.

## Supplementary Material

Crystal structure: contains datablock(s) I, global. DOI: 10.1107/S2056989015023336/nr2062sup1.cif

Structure factors: contains datablock(s) I. DOI: 10.1107/S2056989015023336/nr2062Isup2.hkl

**Fig. 1.**
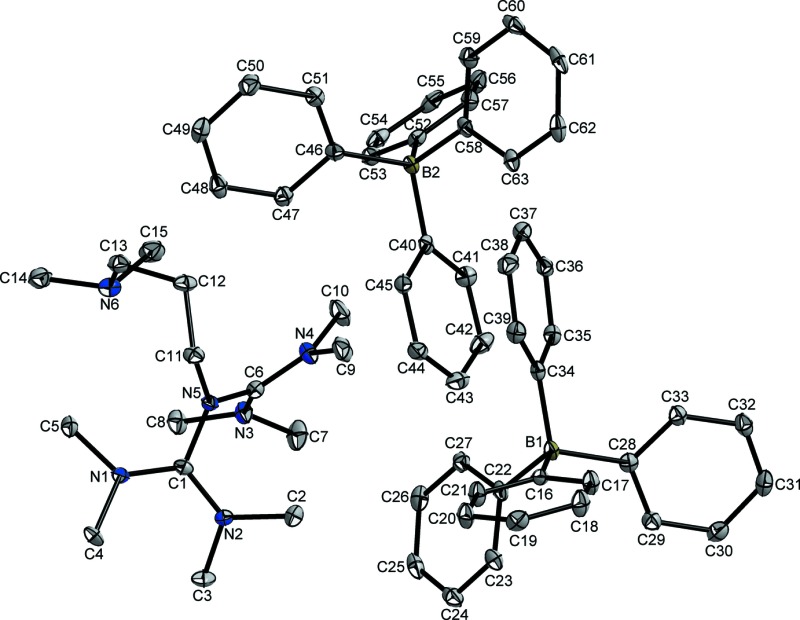
The structure of the title compound with displacement ellipsoids at the 50% probability level. All carbon bonded hydrogen atoms were omitted for the sake of clarity.

. DOI: 10.1107/S2056989015023336/nr2062fig1.tif
The structure of the title compound with displacement ellipsoids at the 50% probability level. All carbon bonded hydrogen atoms were omitted for the sake of clarity.

**Fig. 2.**
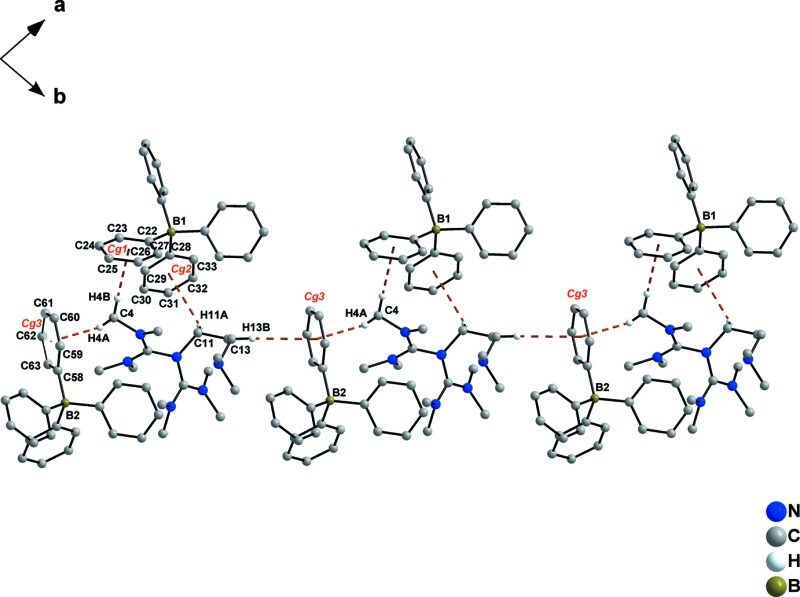
C—H···π interactions (brown dashed lines) between the hydrogen atoms of the guanidinium ion and the phenyl rings (centroids) of the tetraphenylborate ions.

. DOI: 10.1107/S2056989015023336/nr2062fig2.tif
C—H⋯π inter­actions (brown dashed lines) between the hydrogen atoms of the guanidinium ion and the phenyl rings (centroids) of the tetra­phenyl­borate ions.

CCDC reference: 822198

Additional supporting information:  crystallographic information; 3D view; checkCIF report

## Figures and Tables

**Table 1:** Hydrogen-bond geometry (Å, °) *Cg*1, *Cg*2 and *Cg*3 are the centroids of the C22–C27, C28–C33 and C58–C63 rings, respectively.

*D*—H⋯*A*	*D*—H	H⋯*A*	*D*⋯*A*	*D*—H⋯*A*
C4—H4*B*⋯*Cg*1	0.98	2.63	3.597 (2)	171
C4—H4*A*⋯*Cg*3	0.98	2.64	3.580 (2)	161
C11—H11*A*⋯*Cg*2	0.99	2.94	3.500 (2)	117
C13—H13*B*⋯*Cg*3	0.99	2.97	3.950 (2)	170

## supplementary crystallographic information

### S1. Comment

ω-Aminoalkylguanidines like *N*''-[3-(dimethylamino)propyl]- *N*,*N*,*N*',*N*'-tetramethylguanidine (I) (Tiritiris & Kantlehner, 2012*b*), in which two nitrogen atoms with different basicity are present, are considered as ambident nucleophiles. Electrophiles can attack at both, on the imine nitrogen of the guanidine function, as well as on the nitrogen atom of the (dimethylamino)propyl group. By reaction of (I) with one equivalent *N*,*N*,*N*',*N*'- tetramethylchloroformamidinium chloride (Tiritiris & Kantlehner, 2008), the hygroscopic bisamidinium dichloride (II) was formed exclusively. As expected, the reaction occurred preferably at the imine nitrogen of the guanidine function because it is the most basic site. Two similar bisamidinium dichlorides are known in the literature. They where obtained by reaction of *N*,*N*,*N*',*N*'- tetramethylchloroformamidinium chloride (Tiritiris & Kantlehner, 2008) with *N*,*N*,*N*',*N*',*N*''-pentamethylguanidine or *N*-phenyl-*N*',*N*',*N*'',*N*''-tetramethylguanidine, respectively (Bauer *et al.*, 1968). In our case, by anion exchange with sodium tetraphenylborate it was possible to obtain a stable, non-hygroscopic salt. The here presented crystal structure is the first structural study of a dicationic nonasubstituted bisamidinium salt. The asymmetric unit contains one twofold charged cation and two tetraphenylborate ions (Fig. 1). Prominent bond parameters in the bisamidinium ion are: N5–C6 = 1.388 (3) Å, N5–C1 = 1.407 (3) Å, N5–C11 = 1.506 (3) Å, indicating N–C single- and double-bond character of the central C_3_N unit. The C–N–C angles are 117.51 (17)°, 118.92 (17)° and 123.16 (17)°, indicating a nearly ideal trigonal-planar surrounding of the central nitrogen atom N5 by the carbon atoms C1, C6 and C11. The carbon atoms C1 and C6 are further surrounded by the nitrogen atoms N1, N2, N3 and N4. Here, the C–N bonds show double-bond character and the bond lengths range from 1.319 (3) Å to 1.333 (3) Å. The N–C–N angles range from 118.24 (19)° to 121.87 (19)°, indicating again nearly ideal trigonal-planar surroundings of both carbon centres by the nitrogen atoms. The positive charges are delocalized in the planes N1/C1/N2, N3/C6/N4 and C1/N5/C6. The dihedral angle between the plane N1/C1/N2 and N3/C6/N4 is 70.5 (2)°. The bond lengths and angles in both tetraphenylborate ions are in good agreement with the data from the crystal structure analysis of the alkali metal tetraphenylborates (Behrens *et al.*, 2012*a*). C–H···π interactions between the bisamidinium hydrogen atoms of –N(CH_3_)_2_ and –CH_2_ groups and the phenyl carbon atoms [centroids: *Cg*1 = C22–C27, *Cg*2 = C28–C33 and *Cg*3 = C58–C63] of the tetraphenylborate ion are present (Fig. 2), ranging from 2.63 to 2.97 Å (Tab. 1). Such type of C–H···π interactions have been observed in tetraphenylborate salts with pentasubstituted or hexasubstituted guanidinium ions [for example: *N*,*N*,*N*',*N*'- tetramethyl-*N*''-[3-(trimethylazaniumyl)propyl]guanidinium bis(tetraphenylborate) acetone disolvate (Tiritiris, 2013*a*); *N*-[3-(Dimethylamino)propyl]- *N*,*N*',*N*',*N*'',*N*''- pentamethylguanidinium tetraphenylborate (Tiritiris, 2013*b*)]. The phenyl rings form aromatic pockets, in which the cation is embedded. This leads finally to the formation of a two-dimensional supramolecular pattern along the *ab* plane.

### S2. Experimental

The title compound has been obtained by reaction of *N*''-[3-(dimethylamino)propyl]-*N*,*N*,*N*',*N*'-tetramethylguanidine (I) (Tiritiris & Kantlehner, 2012*b*) with one equivalent of *N*,*N*,*N*',*N*'- tetramethylchloroformamidinium chloride (Tiritiris & Kantlehner, 2008) in acetonitrile at room temperature. After evaporation of the solvent the crude *N*-[3-(dimethylamino)propyl]-*N*-(*N*,*N*,*N*',*N*'-tetramethyl-formamidinio)-*N*',*N*',*N*'',*N*''-tetramethyl-guanidinium dichloride (II) was washed with diethylether and dried *in vacuo*. 1.0 g (2.7 mmol) of (II) was dissolved in 20 ml acetonitrile and 1.83 g (5.4 mmol) of sodium tetraphenylborate in 20 ml acetonitrile were added. After stirring for one hour at room temperature, the precipitated sodium chloride was filtered off. The title compound crystallized from a saturated acetonitrile solution after a few months at 273 K, forming colorless single crystals. Yield: 2.15 g (84.9%).

### S3. Refinement

A total number of 34135 reflections have been measured. The data where scaled in the chiral point group *C2* per default by using *SCALEPACK* (Otwinowski & Minor, 1997). After merging all symmetry related reflections and Friedel pairs, a total number of 6569 unique reflections remained (Theta range: 0.41°-28.28°; data completeness: 97.7%; *R*_int_= 0.044). Therefore *R*_int_ given by *SHELXL2014*/7 (Sheldrick, 2015) is meaningless. The data in the *hkl* file used for structure solution and refinement were also scaled in the chiral point group *C2*, but here the Friedel pairs were kept separate. The title compound crystallizes in the non-centrosymmetric space group *Cc*; however, in the absence of significant anomalous scattering effects, the determined Flack parameter *x* = −0.8 (8) (Parsons *et al.*, 2013) is essentially meaningless. The hydrogen atoms of the methyl groups were allowed to rotate with a fixed angle around the C–N bond to best fit the experimental electron density, with *U*_iso_(H) set to 1.5*U*_eq_(C) and d(C—H) = 0.98 Å. The remaining H atoms were placed in calculated positions with d(C—H) = 0.99 Å (H atoms in CH_2_ groups) and (C—H) = 0.95 Å (H atoms in aromatic rings) and refined using a riding model, with *U*_iso_(H) set to 1.2 *U*_eq_(C).

### Crystal data

**Table:**

C_15_H_36_N_6_^2+^·2C_24_H_20_B^−^	*F*(000) = 2024
*M**_r_* = 938.92	*D*_x_ = 1.189 Mg m^−^^3^
Monoclinic, *C**c*	Mo *K*α radiation, λ = 0.71073 Å
*a* = 17.1964 (5) Å	Cell parameters from 6372 reflections
*b* = 17.6641 (7) Å	θ = 0.4–28.3°
*c* = 17.4751 (6) Å	µ = 0.07 mm^−^^1^
β = 98.752 (1)°	*T* = 100 K
*V* = 5246.4 (3) Å^3^	Block, colorless
*Z* = 4	0.22 × 0.18 × 0.15 mm

### Data collection

**Table:**

Bruker–Nonius KappaCCD diffractometer	10531 reflections with *I* > 2σ(*I*)
Radiation source: fine-focus sealed tube	*R*_int_ = 0.040
Graphite monochromator	θ_max_ = 28.2°, θ_min_ = 3.0°
ω scans	*h* = −22→22
11667 measured reflections	*k* = −22→23
11660 independent reflections	*l* = −23→23

### Refinement

**Table:**

Refinement on *F*^2^	Hydrogen site location: inferred from neighbouring sites
Least-squares matrix: full	H-atom parameters constrained
*R*[*F*^2^ > 2σ(*F*^2^)] = 0.038	*w* = 1/[σ^2^(*F*_o_^2^) + (0.0318*P*)^2^ + 3.2124*P*] where *P* = (*F*_o_^2^ + 2*F*_c_^2^)/3
*wR*(*F*^2^) = 0.087	(Δ/σ)_max_ < 0.001
*S* = 1.06	Δρ_max_ = 0.21 e Å^−^^3^
11660 reflections	Δρ_min_ = −0.20 e Å^−^^3^
651 parameters	Extinction correction: *SHELXL2014* (Sheldrick, 2015), Fc^*^=kFc[1+0.001xFc^2^λ^3^/sin(2θ)]^-1/4^
2 restraints	Extinction coefficient: 0.0028 (4)
Primary atom site location: structure-invariant direct methods	Absolute structure: Flack *x* determined using 4294 quotients [(*I*^+^)-(*I*^-^)]/[(*I*^+^)+(*I*^-^)] (Parsons *et al.*, 2013)
Secondary atom site location: difference Fourier map	Absolute structure parameter: −0.8 (8)

### Special details

**Table:**

Geometry. All e.s.d.'s (except the e.s.d. in the dihedral angle between two l.s. planes) are estimated using the full covariance matrix. The cell e.s.d.'s are taken into account individually in the estimation of e.s.d.'s in distances, angles and torsion angles; correlations between e.s.d.'s in cell parameters are only used when they are defined by crystal symmetry. An approximate (isotropic) treatment of cell e.s.d.'s is used for estimating e.s.d.'s involving l.s. planes.
Refinement. Refinement of *F*^2^ against ALL reflections. The weighted *R*-factor *wR* and goodness of fit *S* are based on *F*^2^, conventional *R*-factors *R* are based on *F*, with *F* set to zero for negative *F*^2^. The threshold expression of *F*^2^ > σ(*F*^2^) is used only for calculating *R*-factors(gt) *etc*. and is not relevant to the choice of reflections for refinement. *R*-factors based on *F*^2^ are statistically about twice as large as those based on *F*, and *R*- factors based on ALL data will be even larger.

### Fractional atomic coordinates and isotropic or equivalent isotropic displacement parameters (Å^2^)

**Table:**

	*x*	*y*	*z*	*U*_iso_*/*U*_eq_	
N1	0.54916 (10)	0.40299 (10)	0.24976 (10)	0.0140 (4)	
N2	0.45346 (11)	0.34306 (10)	0.30784 (11)	0.0159 (4)	
N3	0.45873 (11)	0.21150 (10)	0.19896 (11)	0.0166 (4)	
N4	0.53339 (11)	0.14313 (11)	0.29688 (11)	0.0188 (4)	
N5	0.56111 (10)	0.27259 (10)	0.27842 (10)	0.0126 (3)	
N6	0.65631 (11)	0.24425 (11)	0.12663 (11)	0.0174 (4)	
C1	0.52041 (12)	0.34173 (12)	0.27826 (12)	0.0134 (4)	
C2	0.43782 (14)	0.29283 (14)	0.37052 (14)	0.0216 (5)	
H2A	0.4096	0.2478	0.3484	0.032*	
H2B	0.4057	0.3196	0.4036	0.032*	
H2C	0.4877	0.2775	0.4014	0.032*	
C3	0.38691 (14)	0.39186 (14)	0.27671 (15)	0.0241 (5)	
H3A	0.3968	0.4146	0.2280	0.036*	
H3B	0.3808	0.4320	0.3141	0.036*	
H3C	0.3387	0.3616	0.2673	0.036*	
C4	0.52999 (14)	0.47987 (12)	0.27348 (14)	0.0203 (5)	
H4A	0.4923	0.5032	0.2325	0.030*	
H4B	0.5781	0.5104	0.2824	0.030*	
H4C	0.5068	0.4772	0.3213	0.030*	
C5	0.60081 (13)	0.40206 (13)	0.19034 (13)	0.0177 (4)	
H5A	0.6084	0.3497	0.1744	0.027*	
H5B	0.6518	0.4242	0.2114	0.027*	
H5C	0.5767	0.4316	0.1455	0.027*	
C6	0.51696 (12)	0.20783 (12)	0.25854 (12)	0.0141 (4)	
C7	0.38500 (15)	0.16881 (15)	0.19791 (15)	0.0257 (5)	
H7A	0.3841	0.1458	0.2488	0.038*	
H7B	0.3817	0.1290	0.1585	0.038*	
H7C	0.3401	0.2032	0.1858	0.038*	
C8	0.46021 (14)	0.26164 (14)	0.13219 (13)	0.0197 (5)	
H8A	0.4312	0.3082	0.1394	0.030*	
H8B	0.4356	0.2360	0.0849	0.030*	
H8C	0.5148	0.2742	0.1277	0.030*	
C9	0.56851 (15)	0.13745 (14)	0.37887 (14)	0.0242 (5)	
H9A	0.6212	0.1153	0.3828	0.036*	
H9B	0.5354	0.1052	0.4063	0.036*	
H9C	0.5723	0.1880	0.4021	0.036*	
C10	0.51657 (17)	0.06897 (13)	0.25923 (16)	0.0285 (6)	
H10A	0.4707	0.0462	0.2773	0.043*	
H10B	0.5622	0.0356	0.2724	0.043*	
H10C	0.5057	0.0758	0.2030	0.043*	
C11	0.64786 (12)	0.27091 (13)	0.30879 (13)	0.0150 (4)	
H11A	0.6544	0.2568	0.3642	0.018*	
H11B	0.6686	0.3229	0.3058	0.018*	
C12	0.69866 (13)	0.21784 (13)	0.26843 (13)	0.0188 (5)	
H12A	0.6704	0.1691	0.2591	0.023*	
H12B	0.7478	0.2077	0.3045	0.023*	
C13	0.72112 (13)	0.24477 (13)	0.19158 (13)	0.0186 (4)	
H13A	0.7421	0.2969	0.1984	0.022*	
H13B	0.7639	0.2120	0.1783	0.022*	
C14	0.68101 (15)	0.28608 (14)	0.06149 (14)	0.0227 (5)	
H14A	0.7288	0.2630	0.0476	0.034*	
H14B	0.6917	0.3389	0.0766	0.034*	
H14C	0.6390	0.2842	0.0169	0.034*	
C15	0.63482 (15)	0.16667 (14)	0.10226 (15)	0.0250 (5)	
H15A	0.6812	0.1403	0.0892	0.038*	
H15B	0.5938	0.1679	0.0567	0.038*	
H15C	0.6151	0.1399	0.1445	0.038*	
B1	0.29633 (14)	−0.00569 (13)	0.41254 (14)	0.0131 (5)	
C16	0.33345 (12)	0.05140 (12)	0.48320 (13)	0.0135 (4)	
C17	0.34030 (14)	0.03221 (13)	0.56111 (13)	0.0189 (5)	
H17	0.3211	−0.0156	0.5746	0.023*	
C18	0.37429 (15)	0.08026 (13)	0.62042 (13)	0.0214 (5)	
H18	0.3786	0.0645	0.6729	0.026*	
C19	0.40168 (14)	0.15091 (13)	0.60279 (13)	0.0187 (5)	
H19	0.4238	0.1844	0.6428	0.022*	
C20	0.39638 (14)	0.17187 (13)	0.52606 (13)	0.0204 (5)	
H20	0.4155	0.2199	0.5130	0.024*	
C21	0.36316 (14)	0.12288 (13)	0.46788 (13)	0.0190 (5)	
H21	0.3605	0.1385	0.4155	0.023*	
C22	0.24858 (13)	0.04641 (12)	0.34214 (13)	0.0143 (4)	
C23	0.19759 (13)	0.10448 (12)	0.35885 (14)	0.0173 (4)	
H23	0.1922	0.1133	0.4114	0.021*	
C24	0.15475 (14)	0.14951 (13)	0.30210 (14)	0.0200 (5)	
H24	0.1208	0.1877	0.3162	0.024*	
C25	0.16175 (13)	0.13849 (13)	0.22473 (14)	0.0203 (5)	
H25	0.1324	0.1687	0.1856	0.024*	
C26	0.21206 (14)	0.08288 (14)	0.20540 (13)	0.0190 (5)	
H26	0.2178	0.0751	0.1528	0.023*	
C27	0.25439 (13)	0.03814 (12)	0.26329 (13)	0.0156 (4)	
H27	0.2886	0.0004	0.2486	0.019*	
C28	0.23664 (13)	−0.06677 (12)	0.44542 (12)	0.0142 (4)	
C29	0.15612 (13)	−0.05335 (13)	0.44582 (13)	0.0177 (5)	
H29	0.1340	−0.0074	0.4241	0.021*	
C30	0.10721 (14)	−0.10448 (14)	0.47665 (14)	0.0210 (5)	
H30	0.0531	−0.0929	0.4757	0.025*	
C31	0.13767 (14)	−0.17220 (14)	0.50867 (13)	0.0197 (5)	
H31	0.1047	−0.2075	0.5293	0.024*	
C32	0.21682 (14)	−0.18749 (13)	0.50998 (13)	0.0182 (5)	
H32	0.2386	−0.2333	0.5322	0.022*	
C33	0.26451 (14)	−0.13592 (12)	0.47879 (12)	0.0159 (4)	
H33	0.3185	−0.1480	0.4801	0.019*	
C34	0.36745 (13)	−0.05447 (12)	0.38311 (12)	0.0134 (4)	
C35	0.34968 (13)	−0.11383 (13)	0.32968 (13)	0.0151 (4)	
H35	0.2960	−0.1231	0.3096	0.018*	
C36	0.40666 (13)	−0.15960 (12)	0.30481 (12)	0.0157 (4)	
H36	0.3917	−0.1991	0.2686	0.019*	
C37	0.48566 (13)	−0.14740 (13)	0.33310 (13)	0.0187 (5)	
H37	0.5251	−0.1780	0.3160	0.022*	
C38	0.50604 (14)	−0.09022 (14)	0.38638 (14)	0.0204 (5)	
H38	0.5598	−0.0815	0.4064	0.025*	
C39	0.44791 (13)	−0.04539 (13)	0.41076 (13)	0.0169 (4)	
H39	0.4634	−0.0068	0.4479	0.020*	
B2	0.80503 (15)	0.00432 (14)	0.52652 (14)	0.0128 (4)	
C40	0.73194 (13)	0.05104 (12)	0.55564 (11)	0.0134 (4)	
C41	0.65402 (13)	0.02539 (13)	0.54487 (13)	0.0168 (4)	
H41	0.6413	−0.0197	0.5160	0.020*	
C42	0.59422 (14)	0.06346 (15)	0.57499 (13)	0.0217 (5)	
H42	0.5422	0.0438	0.5669	0.026*	
C43	0.61052 (14)	0.12989 (14)	0.61668 (13)	0.0212 (5)	
H43	0.5701	0.1561	0.6372	0.025*	
C44	0.68678 (14)	0.15728 (13)	0.62787 (13)	0.0178 (5)	
H44	0.6988	0.2030	0.6557	0.021*	
C45	0.74583 (13)	0.11816 (13)	0.59849 (12)	0.0157 (4)	
H45	0.7979	0.1377	0.6078	0.019*	
C46	0.86800 (13)	0.06551 (12)	0.49821 (12)	0.0138 (4)	
C47	0.84134 (13)	0.13320 (13)	0.45996 (12)	0.0160 (4)	
H47	0.7866	0.1440	0.4528	0.019*	
C48	0.89142 (14)	0.18477 (13)	0.43229 (13)	0.0185 (5)	
H48	0.8706	0.2297	0.4072	0.022*	
C49	0.97173 (15)	0.17088 (14)	0.44126 (14)	0.0206 (5)	
H49	1.0063	0.2057	0.4221	0.025*	
C50	1.00057 (14)	0.10506 (14)	0.47875 (14)	0.0214 (5)	
H50	1.0553	0.0945	0.4854	0.026*	
C51	0.94928 (13)	0.05445 (13)	0.50663 (13)	0.0174 (4)	
H51	0.9707	0.0102	0.5327	0.021*	
C52	0.76945 (12)	−0.04893 (13)	0.45171 (12)	0.0141 (4)	
C53	0.73887 (13)	−0.01733 (13)	0.37920 (12)	0.0165 (4)	
H53	0.7429	0.0358	0.3723	0.020*	
C54	0.70306 (13)	−0.06078 (14)	0.31746 (13)	0.0187 (5)	
H54	0.6831	−0.0371	0.2697	0.022*	
C55	0.69626 (13)	−0.13857 (14)	0.32521 (13)	0.0195 (5)	
H55	0.6709	−0.1683	0.2834	0.023*	
C56	0.72690 (14)	−0.17218 (13)	0.39466 (13)	0.0188 (5)	
H56	0.7237	−0.2255	0.4005	0.023*	
C57	0.76263 (13)	−0.12778 (13)	0.45626 (13)	0.0165 (4)	
H57	0.7833	−0.1522	0.5034	0.020*	
C58	0.84769 (12)	−0.04918 (12)	0.59834 (13)	0.0133 (4)	
C59	0.90731 (13)	−0.10152 (13)	0.58859 (14)	0.0171 (4)	
H59	0.9228	−0.1061	0.5389	0.021*	
C60	0.94459 (13)	−0.14677 (13)	0.64832 (14)	0.0195 (5)	
H60	0.9848	−0.1809	0.6389	0.023*	
C61	0.92316 (14)	−0.14212 (12)	0.72185 (14)	0.0202 (5)	
H61	0.9489	−0.1724	0.7630	0.024*	
C62	0.86401 (14)	−0.09282 (13)	0.73399 (13)	0.0192 (5)	
H62	0.8482	−0.0895	0.7837	0.023*	
C63	0.82717 (13)	−0.04758 (12)	0.67306 (13)	0.0151 (4)	
H63	0.7864	−0.0143	0.6828	0.018*	

### Atomic displacement parameters (Å^2^)

**Table:**

	*U*^11^	*U*^22^	*U*^33^	*U*^12^	*U*^13^	*U*^23^
N1	0.0133 (8)	0.0109 (8)	0.0181 (9)	0.0019 (7)	0.0032 (7)	−0.0004 (7)
N2	0.0132 (9)	0.0157 (9)	0.0196 (9)	0.0029 (7)	0.0056 (7)	0.0028 (7)
N3	0.0143 (9)	0.0160 (9)	0.0187 (9)	−0.0027 (7)	−0.0005 (7)	0.0019 (7)
N4	0.0214 (10)	0.0139 (9)	0.0207 (10)	0.0006 (7)	0.0022 (8)	0.0039 (7)
N5	0.0113 (8)	0.0114 (8)	0.0148 (8)	0.0014 (7)	0.0012 (7)	0.0012 (7)
N6	0.0162 (9)	0.0182 (9)	0.0173 (9)	0.0019 (7)	0.0015 (7)	−0.0021 (7)
C1	0.0131 (10)	0.0132 (10)	0.0134 (10)	0.0012 (8)	−0.0003 (8)	−0.0013 (8)
C2	0.0234 (12)	0.0217 (12)	0.0223 (12)	0.0010 (9)	0.0115 (10)	0.0040 (9)
C3	0.0144 (11)	0.0249 (12)	0.0337 (13)	0.0080 (9)	0.0056 (9)	0.0059 (10)
C4	0.0251 (12)	0.0089 (10)	0.0277 (12)	0.0014 (9)	0.0067 (10)	−0.0007 (9)
C5	0.0194 (11)	0.0149 (10)	0.0200 (11)	−0.0027 (8)	0.0067 (9)	0.0018 (8)
C6	0.0144 (10)	0.0137 (10)	0.0151 (10)	0.0005 (8)	0.0046 (8)	0.0006 (8)
C7	0.0198 (12)	0.0272 (13)	0.0287 (12)	−0.0119 (10)	−0.0005 (10)	0.0024 (10)
C8	0.0184 (11)	0.0228 (12)	0.0164 (11)	−0.0007 (9)	−0.0018 (9)	0.0051 (9)
C9	0.0275 (13)	0.0219 (12)	0.0224 (12)	0.0030 (10)	0.0014 (10)	0.0095 (9)
C10	0.0381 (15)	0.0126 (11)	0.0346 (14)	−0.0021 (10)	0.0053 (11)	0.0016 (10)
C11	0.0111 (10)	0.0161 (11)	0.0169 (10)	0.0007 (8)	−0.0013 (8)	−0.0001 (8)
C12	0.0143 (10)	0.0204 (11)	0.0209 (11)	0.0062 (8)	0.0006 (8)	0.0007 (9)
C13	0.0146 (10)	0.0191 (11)	0.0222 (11)	0.0027 (8)	0.0028 (9)	−0.0009 (9)
C14	0.0238 (12)	0.0250 (12)	0.0203 (11)	0.0043 (10)	0.0067 (9)	−0.0008 (9)
C15	0.0259 (13)	0.0240 (12)	0.0263 (12)	−0.0019 (10)	0.0074 (10)	−0.0071 (10)
B1	0.0155 (12)	0.0106 (11)	0.0130 (11)	−0.0014 (9)	0.0015 (9)	0.0010 (9)
C16	0.0121 (10)	0.0123 (10)	0.0164 (10)	0.0017 (8)	0.0031 (8)	−0.0006 (8)
C17	0.0255 (12)	0.0142 (10)	0.0173 (11)	−0.0049 (9)	0.0040 (9)	0.0006 (9)
C18	0.0300 (13)	0.0219 (12)	0.0127 (10)	−0.0053 (10)	0.0042 (9)	0.0001 (9)
C19	0.0199 (11)	0.0189 (11)	0.0179 (11)	−0.0038 (9)	0.0042 (9)	−0.0071 (9)
C20	0.0240 (12)	0.0149 (11)	0.0226 (12)	−0.0072 (9)	0.0043 (9)	0.0003 (9)
C21	0.0240 (12)	0.0186 (11)	0.0144 (10)	−0.0044 (9)	0.0033 (9)	0.0009 (8)
C22	0.0147 (10)	0.0114 (10)	0.0166 (10)	−0.0042 (8)	0.0020 (8)	0.0009 (8)
C23	0.0198 (11)	0.0118 (10)	0.0201 (11)	−0.0026 (8)	0.0022 (9)	−0.0005 (8)
C24	0.0179 (11)	0.0123 (11)	0.0291 (12)	−0.0011 (9)	0.0010 (9)	0.0012 (9)
C25	0.0167 (11)	0.0171 (11)	0.0249 (12)	−0.0049 (9)	−0.0041 (9)	0.0096 (9)
C26	0.0188 (11)	0.0226 (12)	0.0154 (10)	−0.0057 (9)	0.0017 (8)	0.0039 (9)
C27	0.0148 (10)	0.0142 (10)	0.0177 (11)	−0.0029 (8)	0.0025 (8)	0.0009 (8)
C28	0.0180 (11)	0.0131 (10)	0.0114 (10)	−0.0012 (8)	0.0015 (8)	−0.0017 (8)
C29	0.0208 (11)	0.0148 (11)	0.0181 (11)	0.0002 (9)	0.0050 (9)	−0.0002 (8)
C30	0.0175 (11)	0.0241 (12)	0.0227 (12)	−0.0019 (9)	0.0077 (9)	−0.0034 (9)
C31	0.0270 (12)	0.0188 (11)	0.0145 (10)	−0.0087 (9)	0.0067 (9)	−0.0014 (9)
C32	0.0278 (12)	0.0136 (11)	0.0135 (10)	−0.0019 (9)	0.0043 (9)	0.0003 (8)
C33	0.0185 (11)	0.0141 (11)	0.0152 (10)	−0.0005 (8)	0.0030 (8)	0.0003 (8)
C34	0.0161 (10)	0.0120 (10)	0.0122 (10)	−0.0005 (8)	0.0027 (8)	0.0034 (8)
C35	0.0149 (10)	0.0154 (11)	0.0149 (10)	−0.0023 (8)	0.0020 (8)	0.0017 (8)
C36	0.0222 (11)	0.0127 (10)	0.0125 (10)	0.0002 (8)	0.0040 (8)	0.0018 (8)
C37	0.0194 (11)	0.0186 (11)	0.0196 (11)	0.0039 (9)	0.0077 (9)	0.0042 (9)
C38	0.0143 (10)	0.0250 (12)	0.0221 (11)	−0.0005 (9)	0.0029 (8)	0.0029 (9)
C39	0.0185 (11)	0.0170 (11)	0.0151 (10)	−0.0028 (8)	0.0023 (8)	−0.0008 (8)
B2	0.0141 (11)	0.0102 (11)	0.0140 (11)	−0.0010 (9)	0.0019 (9)	0.0002 (9)
C40	0.0169 (10)	0.0146 (10)	0.0083 (9)	0.0010 (8)	0.0014 (8)	0.0033 (8)
C41	0.0165 (11)	0.0192 (11)	0.0140 (10)	0.0006 (9)	0.0005 (8)	−0.0011 (8)
C42	0.0144 (11)	0.0321 (13)	0.0186 (11)	0.0000 (9)	0.0020 (9)	−0.0005 (10)
C43	0.0204 (12)	0.0287 (13)	0.0154 (11)	0.0097 (10)	0.0053 (9)	0.0022 (9)
C44	0.0260 (12)	0.0152 (11)	0.0126 (10)	0.0039 (9)	0.0045 (9)	0.0001 (8)
C45	0.0183 (11)	0.0149 (11)	0.0143 (10)	−0.0014 (8)	0.0042 (8)	0.0013 (8)
C46	0.0163 (10)	0.0124 (10)	0.0128 (10)	−0.0012 (8)	0.0023 (8)	−0.0028 (8)
C47	0.0176 (11)	0.0165 (11)	0.0137 (10)	−0.0002 (8)	0.0024 (8)	−0.0009 (8)
C48	0.0267 (12)	0.0158 (11)	0.0128 (10)	−0.0023 (9)	0.0022 (9)	0.0008 (8)
C49	0.0240 (12)	0.0208 (12)	0.0185 (11)	−0.0080 (9)	0.0082 (9)	−0.0012 (9)
C50	0.0172 (11)	0.0218 (12)	0.0262 (12)	−0.0030 (9)	0.0062 (9)	−0.0020 (10)
C51	0.0183 (11)	0.0140 (10)	0.0199 (11)	0.0008 (8)	0.0032 (9)	0.0004 (8)
C52	0.0116 (10)	0.0172 (11)	0.0145 (10)	−0.0017 (8)	0.0046 (8)	−0.0016 (8)
C53	0.0143 (10)	0.0190 (11)	0.0167 (11)	0.0006 (8)	0.0038 (8)	0.0006 (9)
C54	0.0143 (10)	0.0290 (13)	0.0128 (10)	0.0013 (9)	0.0025 (8)	−0.0011 (9)
C55	0.0151 (11)	0.0294 (13)	0.0152 (10)	−0.0055 (9)	0.0059 (8)	−0.0080 (9)
C56	0.0207 (11)	0.0181 (11)	0.0189 (11)	−0.0063 (9)	0.0070 (9)	−0.0044 (9)
C57	0.0187 (11)	0.0169 (11)	0.0143 (10)	−0.0029 (8)	0.0041 (8)	0.0007 (8)
C58	0.0138 (10)	0.0089 (10)	0.0167 (10)	−0.0043 (8)	0.0003 (8)	−0.0007 (8)
C59	0.0169 (11)	0.0145 (11)	0.0198 (11)	−0.0028 (9)	0.0020 (9)	−0.0022 (9)
C60	0.0159 (11)	0.0091 (10)	0.0319 (13)	−0.0014 (8)	−0.0013 (9)	−0.0003 (9)
C61	0.0227 (12)	0.0112 (10)	0.0235 (12)	−0.0068 (9)	−0.0072 (9)	0.0056 (9)
C62	0.0246 (12)	0.0165 (11)	0.0156 (10)	−0.0087 (9)	−0.0004 (9)	0.0026 (9)
C63	0.0157 (10)	0.0129 (10)	0.0157 (10)	−0.0045 (8)	−0.0004 (8)	0.0003 (8)

### Geometric parameters (Å, º)

**Table:**

N1—C1	1.319 (3)	C24—H24	0.9500
N1—C5	1.465 (3)	C25—C26	1.384 (4)
N1—C4	1.472 (3)	C25—H25	0.9500
N2—C1	1.332 (3)	C26—C27	1.398 (3)
N2—C2	1.466 (3)	C26—H26	0.9500
N2—C3	1.469 (3)	C27—H27	0.9500
N3—C6	1.332 (3)	C28—C29	1.406 (3)
N3—C8	1.468 (3)	C28—C33	1.406 (3)
N3—C7	1.473 (3)	C29—C30	1.397 (3)
N4—C6	1.333 (3)	C29—H29	0.9500
N4—C9	1.471 (3)	C30—C31	1.389 (3)
N4—C10	1.475 (3)	C30—H30	0.9500
N5—C6	1.388 (3)	C31—C32	1.384 (3)
N5—C1	1.407 (3)	C31—H31	0.9500
N5—C11	1.506 (3)	C32—C33	1.391 (3)
N6—C13	1.465 (3)	C32—H32	0.9500
N6—C15	1.466 (3)	C33—H33	0.9500
N6—C14	1.473 (3)	C34—C39	1.404 (3)
C2—H2A	0.9800	C34—C35	1.406 (3)
C2—H2B	0.9800	C35—C36	1.390 (3)
C2—H2C	0.9800	C35—H35	0.9500
C3—H3A	0.9800	C36—C37	1.390 (3)
C3—H3B	0.9800	C36—H36	0.9500
C3—H3C	0.9800	C37—C38	1.382 (3)
C4—H4A	0.9800	C37—H37	0.9500
C4—H4B	0.9800	C38—C39	1.392 (3)
C4—H4C	0.9800	C38—H38	0.9500
C5—H5A	0.9800	C39—H39	0.9500
C5—H5B	0.9800	B2—C40	1.648 (3)
C5—H5C	0.9800	B2—C52	1.651 (3)
C7—H7A	0.9800	B2—C58	1.651 (3)
C7—H7B	0.9800	B2—C46	1.658 (3)
C7—H7C	0.9800	C40—C41	1.400 (3)
C8—H8A	0.9800	C40—C45	1.403 (3)
C8—H8B	0.9800	C41—C42	1.397 (3)
C8—H8C	0.9800	C41—H41	0.9500
C9—H9A	0.9800	C42—C43	1.387 (3)
C9—H9B	0.9800	C42—H42	0.9500
C9—H9C	0.9800	C43—C44	1.384 (3)
C10—H10A	0.9800	C43—H43	0.9500
C10—H10B	0.9800	C44—C45	1.390 (3)
C10—H10C	0.9800	C44—H44	0.9500
C11—C12	1.525 (3)	C45—H45	0.9500
C11—H11A	0.9900	C46—C51	1.397 (3)
C11—H11B	0.9900	C46—C47	1.412 (3)
C12—C13	1.529 (3)	C47—C48	1.390 (3)
C12—H12A	0.9900	C47—H47	0.9500
C12—H12B	0.9900	C48—C49	1.388 (4)
C13—H13A	0.9900	C48—H48	0.9500
C13—H13B	0.9900	C49—C50	1.389 (4)
C14—H14A	0.9800	C49—H49	0.9500
C14—H14B	0.9800	C50—C51	1.395 (3)
C14—H14C	0.9800	C50—H50	0.9500
C15—H15A	0.9800	C51—H51	0.9500
C15—H15B	0.9800	C52—C57	1.401 (3)
C15—H15C	0.9800	C52—C53	1.411 (3)
B1—C34	1.641 (3)	C53—C54	1.389 (3)
B1—C16	1.646 (3)	C53—H53	0.9500
B1—C28	1.651 (3)	C54—C55	1.387 (3)
B1—C22	1.651 (3)	C54—H54	0.9500
C16—C17	1.390 (3)	C55—C56	1.381 (3)
C16—C21	1.403 (3)	C55—H55	0.9500
C17—C18	1.397 (3)	C56—C57	1.397 (3)
C17—H17	0.9500	C56—H56	0.9500
C18—C19	1.385 (3)	C57—H57	0.9500
C18—H18	0.9500	C58—C63	1.404 (3)
C19—C20	1.381 (3)	C58—C59	1.410 (3)
C19—H19	0.9500	C59—C60	1.391 (3)
C20—C21	1.390 (3)	C59—H59	0.9500
C20—H20	0.9500	C60—C61	1.392 (4)
C21—H21	0.9500	C60—H60	0.9500
C22—C27	1.404 (3)	C61—C62	1.380 (4)
C22—C23	1.409 (3)	C61—H61	0.9500
C23—C24	1.392 (3)	C62—C63	1.403 (3)
C23—H23	0.9500	C62—H62	0.9500
C24—C25	1.389 (4)	C63—H63	0.9500
			
C1—N1—C5	124.12 (18)	C24—C23—C22	123.2 (2)
C1—N1—C4	122.54 (18)	C24—C23—H23	118.4
C5—N1—C4	113.31 (18)	C22—C23—H23	118.4
C1—N2—C2	123.16 (19)	C25—C24—C23	119.9 (2)
C1—N2—C3	122.49 (19)	C25—C24—H24	120.0
C2—N2—C3	114.24 (19)	C23—C24—H24	120.0
C6—N3—C8	123.28 (18)	C26—C25—C24	119.1 (2)
C6—N3—C7	121.89 (19)	C26—C25—H25	120.4
C8—N3—C7	114.71 (18)	C24—C25—H25	120.4
C6—N4—C9	124.90 (19)	C25—C26—C27	120.0 (2)
C6—N4—C10	121.67 (19)	C25—C26—H26	120.0
C9—N4—C10	113.42 (19)	C27—C26—H26	120.0
C6—N5—C1	117.51 (17)	C26—C27—C22	123.0 (2)
C6—N5—C11	123.16 (17)	C26—C27—H27	118.5
C1—N5—C11	118.92 (17)	C22—C27—H27	118.5
C13—N6—C15	111.06 (18)	C29—C28—C33	114.7 (2)
C13—N6—C14	108.72 (18)	C29—C28—B1	123.8 (2)
C15—N6—C14	109.52 (18)	C33—C28—B1	121.42 (19)
N1—C1—N2	121.87 (19)	C30—C29—C28	123.0 (2)
N1—C1—N5	119.87 (19)	C30—C29—H29	118.5
N2—C1—N5	118.26 (19)	C28—C29—H29	118.5
N2—C2—H2A	109.5	C31—C30—C29	120.0 (2)
N2—C2—H2B	109.5	C31—C30—H30	120.0
H2A—C2—H2B	109.5	C29—C30—H30	120.0
N2—C2—H2C	109.5	C32—C31—C30	119.0 (2)
H2A—C2—H2C	109.5	C32—C31—H31	120.5
H2B—C2—H2C	109.5	C30—C31—H31	120.5
N2—C3—H3A	109.5	C31—C32—C33	120.2 (2)
N2—C3—H3B	109.5	C31—C32—H32	119.9
H3A—C3—H3B	109.5	C33—C32—H32	119.9
N2—C3—H3C	109.5	C32—C33—C28	123.2 (2)
H3A—C3—H3C	109.5	C32—C33—H33	118.4
H3B—C3—H3C	109.5	C28—C33—H33	118.4
N1—C4—H4A	109.5	C39—C34—C35	114.7 (2)
N1—C4—H4B	109.5	C39—C34—B1	125.07 (19)
H4A—C4—H4B	109.5	C35—C34—B1	120.16 (19)
N1—C4—H4C	109.5	C36—C35—C34	123.3 (2)
H4A—C4—H4C	109.5	C36—C35—H35	118.4
H4B—C4—H4C	109.5	C34—C35—H35	118.4
N1—C5—H5A	109.5	C37—C36—C35	119.7 (2)
N1—C5—H5B	109.5	C37—C36—H36	120.1
H5A—C5—H5B	109.5	C35—C36—H36	120.1
N1—C5—H5C	109.5	C38—C37—C36	119.1 (2)
H5A—C5—H5C	109.5	C38—C37—H37	120.4
H5B—C5—H5C	109.5	C36—C37—H37	120.4
N3—C6—N4	120.8 (2)	C37—C38—C39	120.1 (2)
N3—C6—N5	118.24 (19)	C37—C38—H38	119.9
N4—C6—N5	120.99 (19)	C39—C38—H38	119.9
N3—C7—H7A	109.5	C38—C39—C34	123.1 (2)
N3—C7—H7B	109.5	C38—C39—H39	118.5
H7A—C7—H7B	109.5	C34—C39—H39	118.5
N3—C7—H7C	109.5	C40—B2—C52	108.77 (18)
H7A—C7—H7C	109.5	C40—B2—C58	108.87 (18)
H7B—C7—H7C	109.5	C52—B2—C58	110.12 (17)
N3—C8—H8A	109.5	C40—B2—C46	109.23 (17)
N3—C8—H8B	109.5	C52—B2—C46	108.39 (17)
H8A—C8—H8B	109.5	C58—B2—C46	111.42 (18)
N3—C8—H8C	109.5	C41—C40—C45	115.3 (2)
H8A—C8—H8C	109.5	C41—C40—B2	123.78 (19)
H8B—C8—H8C	109.5	C45—C40—B2	120.80 (19)
N4—C9—H9A	109.5	C42—C41—C40	122.5 (2)
N4—C9—H9B	109.5	C42—C41—H41	118.7
H9A—C9—H9B	109.5	C40—C41—H41	118.7
N4—C9—H9C	109.5	C43—C42—C41	120.2 (2)
H9A—C9—H9C	109.5	C43—C42—H42	119.9
H9B—C9—H9C	109.5	C41—C42—H42	119.9
N4—C10—H10A	109.5	C44—C43—C42	118.8 (2)
N4—C10—H10B	109.5	C44—C43—H43	120.6
H10A—C10—H10B	109.5	C42—C43—H43	120.6
N4—C10—H10C	109.5	C43—C44—C45	120.3 (2)
H10A—C10—H10C	109.5	C43—C44—H44	119.9
H10B—C10—H10C	109.5	C45—C44—H44	119.9
N5—C11—C12	117.10 (18)	C44—C45—C40	122.9 (2)
N5—C11—H11A	108.0	C44—C45—H45	118.6
C12—C11—H11A	108.0	C40—C45—H45	118.6
N5—C11—H11B	108.0	C51—C46—C47	114.6 (2)
C12—C11—H11B	108.0	C51—C46—B2	124.47 (19)
H11A—C11—H11B	107.3	C47—C46—B2	120.90 (19)
C11—C12—C13	117.00 (19)	C48—C47—C46	123.1 (2)
C11—C12—H12A	108.0	C48—C47—H47	118.5
C13—C12—H12A	108.0	C46—C47—H47	118.5
C11—C12—H12B	108.0	C49—C48—C47	120.2 (2)
C13—C12—H12B	108.0	C49—C48—H48	119.9
H12A—C12—H12B	107.3	C47—C48—H48	119.9
N6—C13—C12	114.50 (19)	C48—C49—C50	118.6 (2)
N6—C13—H13A	108.6	C48—C49—H49	120.7
C12—C13—H13A	108.6	C50—C49—H49	120.7
N6—C13—H13B	108.6	C49—C50—C51	120.1 (2)
C12—C13—H13B	108.6	C49—C50—H50	119.9
H13A—C13—H13B	107.6	C51—C50—H50	119.9
N6—C14—H14A	109.5	C50—C51—C46	123.3 (2)
N6—C14—H14B	109.5	C50—C51—H51	118.3
H14A—C14—H14B	109.5	C46—C51—H51	118.3
N6—C14—H14C	109.5	C57—C52—C53	114.92 (19)
H14A—C14—H14C	109.5	C57—C52—B2	123.07 (19)
H14B—C14—H14C	109.5	C53—C52—B2	121.89 (19)
N6—C15—H15A	109.5	C54—C53—C52	122.6 (2)
N6—C15—H15B	109.5	C54—C53—H53	118.7
H15A—C15—H15B	109.5	C52—C53—H53	118.7
N6—C15—H15C	109.5	C55—C54—C53	120.3 (2)
H15A—C15—H15C	109.5	C55—C54—H54	119.8
H15B—C15—H15C	109.5	C53—C54—H54	119.8
C34—B1—C16	109.53 (18)	C56—C55—C54	119.0 (2)
C34—B1—C28	107.35 (17)	C56—C55—H55	120.5
C16—B1—C28	109.26 (18)	C54—C55—H55	120.5
C34—B1—C22	111.64 (18)	C55—C56—C57	120.0 (2)
C16—B1—C22	108.03 (17)	C55—C56—H56	120.0
C28—B1—C22	111.00 (18)	C57—C56—H56	120.0
C17—C16—C21	115.3 (2)	C56—C57—C52	123.1 (2)
C17—C16—B1	123.41 (19)	C56—C57—H57	118.5
C21—C16—B1	121.27 (19)	C52—C57—H57	118.5
C16—C17—C18	122.8 (2)	C63—C58—C59	114.6 (2)
C16—C17—H17	118.6	C63—C58—B2	123.37 (19)
C18—C17—H17	118.6	C59—C58—B2	122.0 (2)
C19—C18—C17	120.0 (2)	C60—C59—C58	123.1 (2)
C19—C18—H18	120.0	C60—C59—H59	118.4
C17—C18—H18	120.0	C58—C59—H59	118.4
C20—C19—C18	118.9 (2)	C59—C60—C61	120.1 (2)
C20—C19—H19	120.6	C59—C60—H60	119.9
C18—C19—H19	120.6	C61—C60—H60	119.9
C19—C20—C21	120.2 (2)	C62—C61—C60	118.9 (2)
C19—C20—H20	119.9	C62—C61—H61	120.5
C21—C20—H20	119.9	C60—C61—H61	120.5
C20—C21—C16	122.8 (2)	C61—C62—C63	120.1 (2)
C20—C21—H21	118.6	C61—C62—H62	120.0
C16—C21—H21	118.6	C63—C62—H62	120.0
C27—C22—C23	114.8 (2)	C62—C63—C58	123.1 (2)
C27—C22—B1	124.9 (2)	C62—C63—H63	118.5
C23—C22—B1	120.40 (19)	C58—C63—H63	118.5
			
C5—N1—C1—N2	−154.0 (2)	B1—C28—C33—C32	177.3 (2)
C4—N1—C1—N2	24.1 (3)	C16—B1—C34—C39	−3.6 (3)
C5—N1—C1—N5	26.5 (3)	C28—B1—C34—C39	−122.2 (2)
C4—N1—C1—N5	−155.43 (19)	C22—B1—C34—C39	116.0 (2)
C2—N2—C1—N1	−149.4 (2)	C16—B1—C34—C35	172.12 (19)
C3—N2—C1—N1	34.7 (3)	C28—B1—C34—C35	53.6 (3)
C2—N2—C1—N5	30.1 (3)	C22—B1—C34—C35	−68.3 (2)
C3—N2—C1—N5	−145.8 (2)	C39—C34—C35—C36	−0.9 (3)
C6—N5—C1—N1	−138.3 (2)	B1—C34—C35—C36	−177.11 (19)
C11—N5—C1—N1	48.8 (3)	C34—C35—C36—C37	−0.1 (3)
C6—N5—C1—N2	42.1 (3)	C35—C36—C37—C38	0.7 (3)
C11—N5—C1—N2	−130.7 (2)	C36—C37—C38—C39	−0.4 (3)
C8—N3—C6—N4	−147.8 (2)	C37—C38—C39—C34	−0.7 (3)
C7—N3—C6—N4	36.5 (3)	C35—C34—C39—C38	1.3 (3)
C8—N3—C6—N5	30.7 (3)	B1—C34—C39—C38	177.3 (2)
C7—N3—C6—N5	−145.0 (2)	C52—B2—C40—C41	26.9 (3)
C9—N4—C6—N3	−151.7 (2)	C58—B2—C40—C41	−93.1 (2)
C10—N4—C6—N3	27.8 (3)	C46—B2—C40—C41	145.0 (2)
C9—N4—C6—N5	29.8 (3)	C52—B2—C40—C45	−157.59 (19)
C10—N4—C6—N5	−150.7 (2)	C58—B2—C40—C45	82.4 (2)
C1—N5—C6—N3	42.4 (3)	C46—B2—C40—C45	−39.5 (3)
C11—N5—C6—N3	−145.0 (2)	C45—C40—C41—C42	−0.4 (3)
C1—N5—C6—N4	−139.1 (2)	B2—C40—C41—C42	175.3 (2)
C11—N5—C6—N4	33.5 (3)	C40—C41—C42—C43	0.8 (3)
C6—N5—C11—C12	43.4 (3)	C41—C42—C43—C44	−0.2 (3)
C1—N5—C11—C12	−144.2 (2)	C42—C43—C44—C45	−0.8 (3)
N5—C11—C12—C13	78.9 (3)	C43—C44—C45—C40	1.2 (3)
C15—N6—C13—C12	−71.4 (2)	C41—C40—C45—C44	−0.6 (3)
C14—N6—C13—C12	168.05 (19)	B2—C40—C45—C44	−176.4 (2)
C11—C12—C13—N6	−72.2 (3)	C40—B2—C46—C51	146.5 (2)
C34—B1—C16—C17	−92.8 (2)	C52—B2—C46—C51	−95.1 (2)
C28—B1—C16—C17	24.5 (3)	C58—B2—C46—C51	26.2 (3)
C22—B1—C16—C17	145.4 (2)	C40—B2—C46—C47	−36.0 (3)
C34—B1—C16—C21	84.9 (2)	C52—B2—C46—C47	82.3 (2)
C28—B1—C16—C21	−157.7 (2)	C58—B2—C46—C47	−156.35 (19)
C22—B1—C16—C21	−36.9 (3)	C51—C46—C47—C48	0.4 (3)
C21—C16—C17—C18	0.0 (3)	B2—C46—C47—C48	−177.3 (2)
B1—C16—C17—C18	177.8 (2)	C46—C47—C48—C49	0.3 (3)
C16—C17—C18—C19	1.1 (4)	C47—C48—C49—C50	−0.5 (3)
C17—C18—C19—C20	−1.5 (4)	C48—C49—C50—C51	−0.1 (3)
C18—C19—C20—C21	0.7 (4)	C49—C50—C51—C46	0.8 (4)
C19—C20—C21—C16	0.4 (4)	C47—C46—C51—C50	−1.0 (3)
C17—C16—C21—C20	−0.7 (3)	B2—C46—C51—C50	176.6 (2)
B1—C16—C21—C20	−178.6 (2)	C40—B2—C52—C57	−106.9 (2)
C34—B1—C22—C27	13.9 (3)	C58—B2—C52—C57	12.3 (3)
C16—B1—C22—C27	134.4 (2)	C46—B2—C52—C57	134.5 (2)
C28—B1—C22—C27	−105.9 (2)	C40—B2—C52—C53	69.0 (3)
C34—B1—C22—C23	−165.91 (19)	C58—B2—C52—C53	−171.79 (19)
C16—B1—C22—C23	−45.4 (3)	C46—B2—C52—C53	−49.7 (3)
C28—B1—C22—C23	74.3 (2)	C57—C52—C53—C54	1.4 (3)
C27—C22—C23—C24	1.3 (3)	B2—C52—C53—C54	−174.8 (2)
B1—C22—C23—C24	−178.9 (2)	C52—C53—C54—C55	−0.2 (3)
C22—C23—C24—C25	−0.5 (3)	C53—C54—C55—C56	−1.2 (3)
C23—C24—C25—C26	−0.5 (3)	C54—C55—C56—C57	1.3 (3)
C24—C25—C26—C27	0.7 (3)	C55—C56—C57—C52	0.0 (4)
C25—C26—C27—C22	0.1 (3)	C53—C52—C57—C56	−1.3 (3)
C23—C22—C27—C26	−1.1 (3)	B2—C52—C57—C56	174.8 (2)
B1—C22—C27—C26	179.1 (2)	C40—B2—C58—C63	−5.3 (3)
C34—B1—C28—C29	−152.8 (2)	C52—B2—C58—C63	−124.4 (2)
C16—B1—C28—C29	88.5 (2)	C46—B2—C58—C63	115.2 (2)
C22—B1—C28—C29	−30.5 (3)	C40—B2—C58—C59	173.59 (19)
C34—B1—C28—C33	30.1 (3)	C52—B2—C58—C59	54.4 (3)
C16—B1—C28—C33	−88.6 (2)	C46—B2—C58—C59	−65.9 (3)
C22—B1—C28—C33	152.4 (2)	C63—C58—C59—C60	−1.6 (3)
C33—C28—C29—C30	−0.2 (3)	B2—C58—C59—C60	179.4 (2)
B1—C28—C29—C30	−177.4 (2)	C58—C59—C60—C61	0.5 (3)
C28—C29—C30—C31	−0.1 (3)	C59—C60—C61—C62	0.8 (3)
C29—C30—C31—C32	0.6 (3)	C60—C61—C62—C63	−0.9 (3)
C30—C31—C32—C33	−0.7 (3)	C61—C62—C63—C58	−0.3 (3)
C31—C32—C33—C28	0.5 (3)	C59—C58—C63—C62	1.5 (3)
C29—C28—C33—C32	0.0 (3)	B2—C58—C63—C62	−179.5 (2)

### Hydrogen-bond geometry (Å, º)

*Cg*1, *Cg*2 and *Cg*3 are the centroids of the C22–C27, C28–C33 and C58–C63 rings, respectively.

**Table:**

*D*—H···*A*	*D*—H	H···*A*	*D*···*A*	*D*—H···*A*
C4—H4*B*···*Cg*1	0.98	2.63	3.597 (2)	171
C4—H4*A*···*Cg*3	0.98	2.64	3.580 (2)	161
C11—H11*A*···*Cg*2	0.99	2.94	3.500 (2)	117
C13—H13*B*···*Cg*3	0.99	2.97	3.950 (2)	170
