# Supplementary figures and images for: Crystal structure of N-[3-(di­methyl­amino)­prop­yl]-N′,N′,N′′,N′′-tetra­methyl-N-(N,N,N′,N′-tetra­methyl­form­amid­in­ium­yl)guanidinium bis­(tetra­phenyl­borate)
Source: Acta Crystallogr E Crystallogr Commun. 2015 Dec 12;71(Pt 12):o1045–6. doi: 10.1107/S2056989015023336 (PMC4719971; doi:10.1107/S2056989015023336)

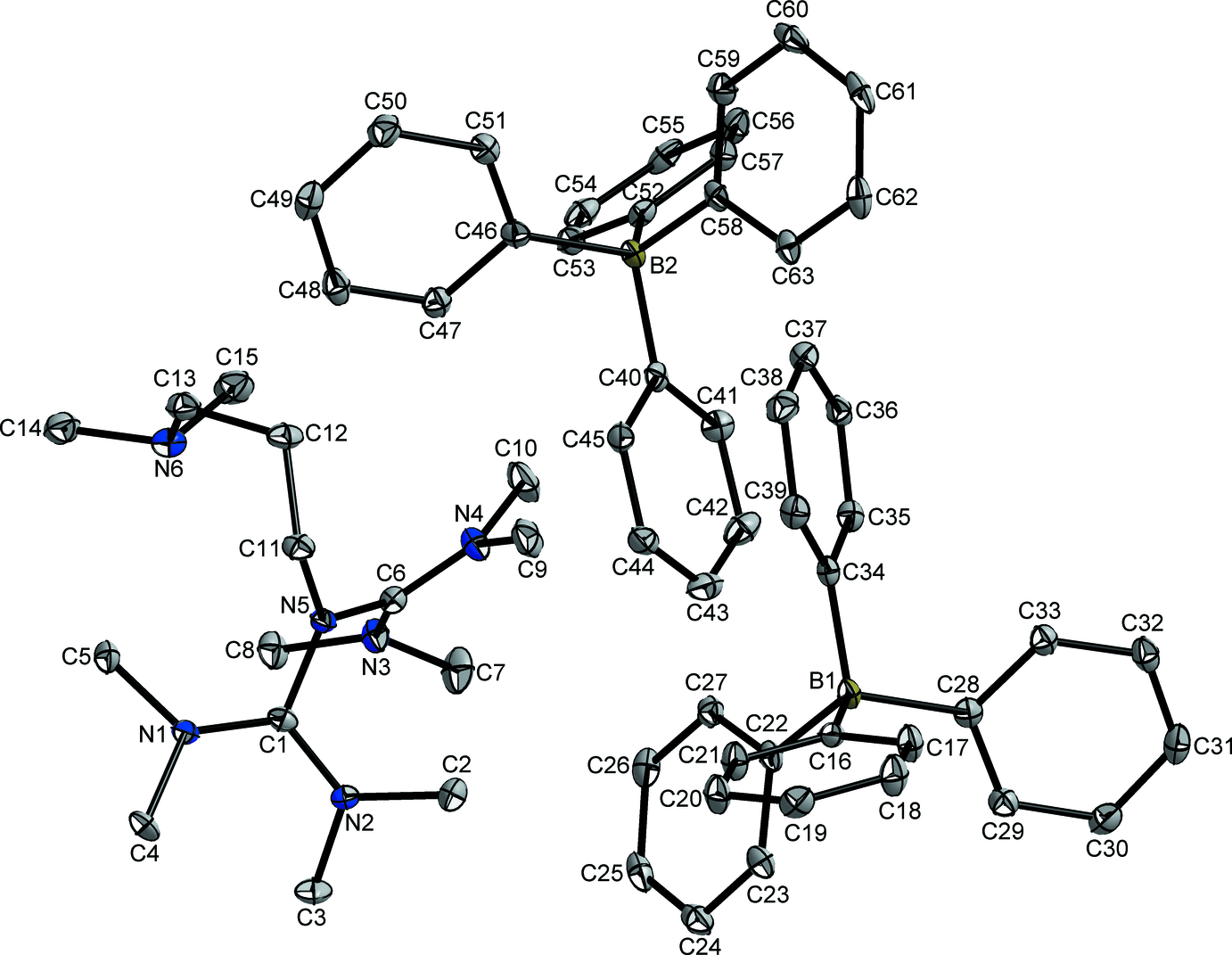

Supplement: Supplementary file 3 [file e-71-o1045-fig1.tif]

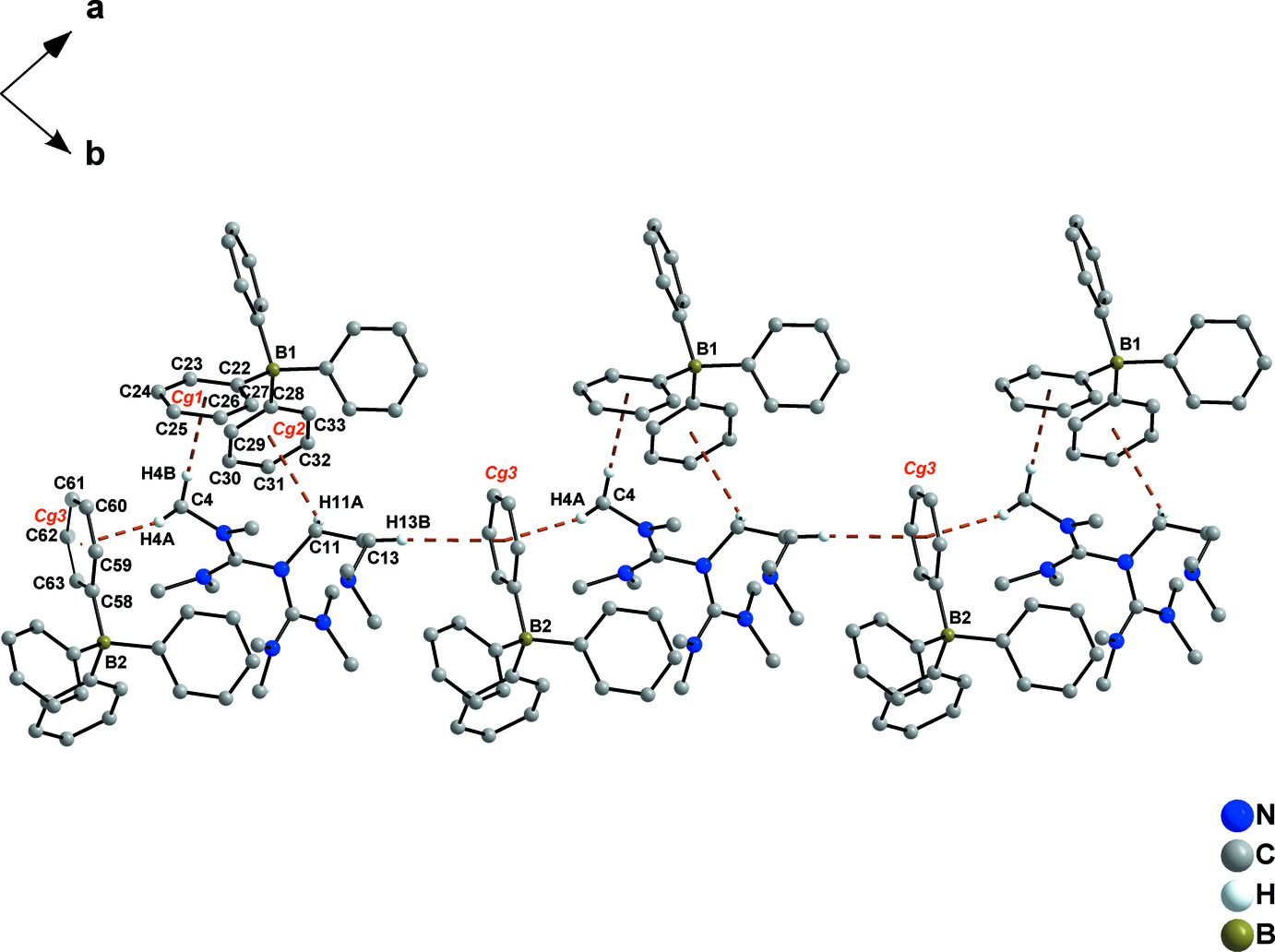

Supplement: Supplementary file 4 [file e-71-o1045-fig2.tif]
